# Generic optimal power flow solution associated with technical improvements and emission reduction by multi-objective ARO algorithm

**DOI:** 10.1038/s41598-025-09976-y

**Published:** 2025-07-22

**Authors:** Amlak Abaza, Ragab A. El-Sehiemy, Zakeria Elbarabry, Asmaa F. Barakat

**Affiliations:** 1 Electrical Engineering Department, Faculty of Engineering, Kafrelsheikh University, Kafrelsheikh, 33516 Egypt; 2 Sustainability Competence Centre, Széchenyi István University, Egyetem square 1, Győr, H-9026 Hungary; 3 Department of Industrial Engineering, King Khalid University, Abha, Saudi Arabia; 4 Electrical Engineering Department, Higher Institute of Engineering and Technology, Kafrelsheikh, Egypt

**Keywords:** Optimal power flow (OPF), Multi-objective ARO, Technical and economic aspects, Environmental concerns, Energy science and technology, Engineering, Mathematics and computing

## Abstract

In modern power engineering, the optimal operation aims to achieve the basic requirements of the electrical power grid, meet various technical and economic aspects, and preserve the environmental limits within their accepted bounds. In this line, the current paper finds the optimal operational scheduling of the power generation units that cover the load requirements, considering different frameworks of the optimal power flow (OPF) problem involving single- and multi-objective functions. Technical, economic, and emissions objective functions are considered. Artificial rabbits’ optimization (ARO) is developed to find the optimal OPF framework solution. The effectiveness of the proposed algorithm is evaluated through a comprehensive comparison study with the existing works in the literature. With six IEEE standard power systems, 22 different cases are implemented to test the ARO performance as an alternative to solve the OPF problem. Two of these systems are considered small-size systems, 30-, and 57-test systems, while the other four are large-scale power systems (IEEE 300, 1354, 3012, and 9241 test systems) to expand the validation scope of this paper. This comparison validates the scalability and efficiency of the ARO algorithm. The impact of varied population size and maximum iteration number is tested for two test systems, the most benchmarking test systems. It was proven that the routine of ARO has robust and superior competitive performance compared with others at fine convergence rates. Significant improvements are acquired in the range of 47% in the technical and economic issues by accepting the environmental concerns.

## Introduction

The economic operating conditions of electrical power systems pose one of the most significant challenges. The key lies in selecting optimal control variables and system quantities. The resolution of optimal power flow (OPF) remains a persistent challenge within the realm of electric power systems. The problem of the OPF in electrical networks is a nonlinear optimization problem that was introduced by Cf. Carpenter in 1962^1^. The OPF has gained significant research attention as an important topic. It involves determining the optimal adjustment of control variables to optimize specific objective functions. OPF addresses the optimization of discrete and continuous control variables, encompassing minimizing of generation fuel costs and active power losses while improving voltage deviation, enhancing voltage stability, and ensuring system security with satisfying both equality and inequality constraints. Consequently, numerous researchers have focused on finding solutions to the OPF problem using both classical and metaheuristic optimization algorithms^2^.

Due to the complexity of the non-convex and non-differentiable objective functions (OFs) encountered in the OPF problem, classical methods such as linear programming, Newton methods, dynamic and interior-point methods have proven inadequate. As a result, metaheuristic optimization algorithms have been developed to efficiently tackle these complexities and attain the global optimum solution^3^. Over the past few decades, metaheuristic algorithms have exhibited successful and extensive application in solving various electric power system problems, including the multi-objective OPF problem^4^. Examples of such metaheuristic optimization algorithms include genetic algorithm (GA)^5^, particle swarm optimization (PSO)^6^, whale optimization algorithm (WOA)^7^, ant colony optimization (ACO)^8^, artificial bee colony (ABC)^9^, gray wolf optimizer (GWO)^10^, Physics-informed neural networks^11^, adaptive differential evolutionary algorithm^12^, firefly algorithm (FA)^13^, moth-flame optimization (MFO)^14^, cuckoo search algorithm^15^, adaptive seeker optimization algorithm^16^, JAYA optimizer^17^, marine predators algorithm (MPA)^18^, hybrid PSO and FA^19^, modified coyote optimization algorithm (MCOA)^20^, salp swarm optimization (SSO) algorithm^21^ and circle search algorithm (CSA)^22^.

Over the past few years, numerous researchers have dedicated their efforts to exploring advanced solutions for the power system’s OPF problem. Some notable studies in this area are outlined below: in^23^, multi-objective firefly algorithm with CPA was suggested for solving the MO-OPF problem. In^24^, DE algorithm was integrated with effective constraint handling techniques are introduced for OPF problem solution. Using AMTPG-JAYA technique, a single-objective OPF was optimized in^25^. Additionally, Table 1 presents improved methods, different objectives functions applied on different systems. This table presents several studies that attempt to reach the best solution to the OPF problem.

**Table 1:** Some metaheuristic algorithms presented recently for solving OPF problem in some literature review.

Ref.	year	Objective functions	Studied systems	Methods
Fuel cost (FC), power loss (PL), voltage deviation (VD) and voltage stability index (VSI)				
^26^	2020	optimization of bi- and tri- objectives of (FC, PL, VD, L-index, voltage profile improvement)	IEEE 30-bus and IEEE 57-bus test systems	Hybrid firefly and PSO algorithm
^27^	2024	optimization of single objectives (FC, PL, produced emission)	IEEE (30-, 57- and 118-) bus systems and practical West Delta Region	Enhanced Social Network Search Technique
^28^	2020	Minimization of multi objectives (FC, emission, PL)	IEEE 30-bus and IEEE 57-bus systems	bat algorithm
^29^	2020	optimization of single objective and multi objective of (FC, PL, VD, L-index, emission)	IEEE (30-, 57- and 118-) bus systems	Hybrid particle swarm and salp optimization algorithm
^30^	2022	single and multi-objective of (FC, PL, VD)	IEEE (14, 30, 39, 57 and 118) -bus test systems.	Whale and Moth-Flame Optimization Algorithms
^31^	2023	Single objective of (FC, emission, L-index, PL)	IEEE 30-bus and IEEE 57-bus systems	Cross-Entropy Method
^32^	2021	Minimization of (FC, emission, PL)	IEEE 30-, 57-, and 118-bus test systems	hybrid self-adaptive heuristic algorithm
^33^	2022	Single and multi-objectives of (FC, emission, PL, VSI)	IEEE 30- and 118-bus test systems	Marine Predators Algorithm
^34^	2023	three single objective functions (FC, VD, PL)	IEEE 30-bus test system	Mountain Gazelle Algorithm
^35^	2023	three single objective functions (FC, VD, PL)	IEEE 57-bus and 118-bus test systems and practical West Delta Region system	enhanced quasi-reflection jellyfish
^36^	2023	single and multi-objective of (FC, PL, VD, emission, L-index)	IEEE 30-, 57-, and 118-bus test systems	Beetle swarm optimization algorithm
^37^	2024	Minimization of single objectives (FC, emission, L-index, PL)	IEEE 30-bus and 118-bus test systems	Arithmetic optimization algorithm and Aquila optimize
^38^	2025	single and multi-objective of (FC, emission)	IEEE 30-bus test system	pelican optimization algorithm
^39^	2022	single objectives (FC, emission, PL)	IEEE 30-bus and 57-bus test systems	Teaching learning-based optimizer
^40^	2022	single objective of (FC, PL, VD, emission, L-index)	IEEE 30-, 57-, and 118-bus test systems	Moth flame optimization algorithm

The research gap in the previously reported methods are the classical deterministic optimization techniques that offer solutions with well-understood convergence properties and analytical foundations. However, these approaches assume differentiability and convexity yield suboptimal or infeasible solutions. The search-based methods have the following challenges: The missing of guaranteed convergence to a global optimum; The effectiveness of the previous methods is dependent on the selection and tuning mechanism of population size, inertia weights, and mutation rates for each algorithm; The random behavior of such algorithms produces limiting reproducibility and consistency; The associated computational burden is affected by the large-scale systems; these metaheuristics generally do not incorporate uncertainty handling mechanisms,

This paper addresses the OPF problem by simultaneously optimizing multiple objectives. These are minimizing the active power loss, fuel cost, emission, voltage deviation at the load buses, and ameliorating voltage stability index (VSI) while accounting for both equality and inequality constraints. In this line, the current paper finds the optimal operational output of power generation units that covers the load requirements considering the artificial rabbits’ optimization (ARO) is employed to find the solution of the considered OPF problem. The effectiveness of the proposed algorithm is evaluated through a comprehensive comparison study with the existing works in literature. Six standard IEEE power systems were also used with 22 different cases studied for testing the ARO performance in solving the OPF problem. Also, this paper extends its validation on different size large-scale test systems standard systems. It was proven that the routine of ARO has robust, and superior competitive performance compared with others at fine convergence rates. Significant technical and economic improvements are acquired.

The significant contribution of this paper can be summarized as follows:

This paper proposes multi-dimension optimal operation solution of the optimal power flow problem using ARO method.

The validation and effectiveness of the proposed algorithm is evaluated on small scale and large-scale IEEE power systems.

Statistical analyses are procced to prove the has robust, and superior competitive performance compared with others at fine convergence rates.

Sensitivity analyses in terms of varied population size and iteration number are also provided for sample systems.

Significant technical, economic and environmental improvements are achieved compared with several previous optimization techniques.

The remaining sections of this paper are structured as follows: Section “Problem formulation” introduces the formulation of the OPF problem. Section “Proposed solution methodology” illustrates the proposed ARO algorithm. In Section “Experimental simulations”, briefly introduces the simulation results, analysis of the experiment and the best compromise using the IEEE test systems. Section “*Numerical simulation applications*” provides a discussion of these results, while Section “Conclusion” serves as the conclusion, summarizing the main research findings and suggesting future trends.

## Problem formulation

The main objective of the OPF problem is to optimize system objectives while adhering to predefined constraints. Solving the OPF leads to the determination of optimal control variable settings. In this context, we introduce a compact formula that facilitates the control of specific research challenges. Mathematically, OPF can be expressed as^41,42^:

### Objectives

The general expression for multiobjective optimization problem can be expressed in the following set of equations, Eqs. (1)-(4), which are :

1 $$\:{min}{f}_{i}\left(x,u\right)=\left\{{f}_{1}\left(x,u\right),{f}_{2}\left(x,u\right),{f}_{3}\left(x,u\right),\dots\:\dots\:.{f}_{n}\left(x,u\right)\right\},\:\:i=\text{1,2},\dots\:\dots\:.,{N}_{obj}$$

where, $\:{f}_{i}\left(x,u\right)$ in Eq. (1) is the objective function to be optimized, with N_obj_ representing the total number of objectives.

The objective functions in Eq. (1) are subjected to operational constraints that are represented in Eqs. (2) and (3). Both constraints are defined in terms of equality and inequality, are expressed as $\:g\left(x,u\right)$ and $\:h\left(x,u\right)$ respectively. Equation (4) represents the lower and upper limits for each of dependent and control variables z (‘x’ and ‘u’), represent vectors of dependent and control variables, respectively^19^

2 $$\:{g}_{i}\left(x,u\right)=0$$

3 $$\:{h}_{i}\left(x,u\right)\le\:0$$

4 $$\:zmin\:<z<zmax$$

The vectors (x) and (u) can be expressed as follows:

5 $$\:{x}^{T}=\left[{P}_{G1},\:{V}_{L1}\dots\:{V}_{{LN}_{pq}},\:{Q}_{G1}\dots\:{Q}_{{G}_{NG}},{S}_{L1}\dots\:{S}_{{L}_{Nl}}\right]$$

6 $$\:{u}^{T}=\left[{P}_{G2}\dots\:.{P}_{{G}_{NG}},\:{V}_{G1}\dots\:{V}_{{G}_{NG}},\:{{T}_{1}\dots\:{T}_{{L}_{Nt}},Q}_{C1}\dots\:{Q}_{{C}_{NC}}\right]$$

where $\:{P}_{G1}$ signifies the real power of the chosen slack bus, while $\:{V}_{{LN}_{pq}}$ denotes the voltage at load buses. $\:{Q}_{{G}_{NG}}$ refers to the generation reactive power. $\:{S}_{{L}_{Nl}}$ denotes the apparent power passes in transmission lines^3^. *V*_*G1*_, $\:{T}_{1}$, *Q*_*C1*_ and *P*_*G2*_ represent the voltage, transformer tapping ratio, reactive/active power generation at PV buses, respectively. *NG*,* NC* and *Nt* stand for the count of generator units, reactive power compensators, and regulating transformers, respectively.

This paper aims to attain three distinct advantages for the power system, categorized as economic, technical, and environmental categories.

#### Economic category

The 1st economic OF in this context seeks to minimize the cumulative fuel cost of the power-generating units, denominated in $/h, as outlined in Eq. (7). The pursuit of economic advantages involves the minimization of fuel costs related to generating units, which is mathematically stated as a function of the active generating power ‘$$\:{P}_{gi}$$’ and the cost coefficients ‘*a*_*i*_, *b*_*i*_ and *c*_*i*_’ of i^th^ generator units^43^.

7 $$\:\text{M}\text{i}\text{n}\:{F}_{1}={\sum\:}_{i=1}^{{N}_{g}}{a}_{i}\text{P}{{\text{g}}_{i}}^{2}+{b}_{i}\text{P}{\text{g}}_{i}+{c}_{i}\text{\$}/\text{h}\text{r}$$

#### Technical category

The technical advantages within power systems encompass the reduction of active power losses, the improvement of voltage stability indices, and the enhancement of the voltage profile through the reduction of the deviation of the voltage at load buses from the target voltages at these buses^3^.

Minimization of active power losses.

The 2nd technical OF is designed to achieve the minimization of power losses P_L_ in all transmission lines ‘*N*_*l*_’, as described in Eq. (8).

8 $$\:{\:Min\:F}_{2}\left(x,u\right)={P}_{L}=\sum\:_{k=1}^{{N}_{l}}\left({G}_{k}\left({V}_{i}^{2}{+V}_{j}^{2}-2{V}_{i}{V}_{j}cos{\delta\:}_{ij}\right)\right)\left(\text{M}\text{W}\right)$$

where, *V*_*i*_ and *V*_*j*_ are the voltages at buses *i* and *j*, $\:{\delta\:}_{ij}$ is the voltage angle variation between bus i and j that connect the line *l* and G_k_ is the conductance of branch k between bus i and j.

Minimization of total voltage deviation.

The 3rd technical OF focuses on reducing voltage deviations $\:\varDelta\:V$ at the load buses $\:{\varvec{N}}_{\varvec{p}\varvec{q}}\:$to improve the voltage profile at these locations, as in Eq. (9).

9 $$\:{\:Min\:F}_{3}\left(x,u\right)=\varDelta\:V=\:\sum\:_{i=1}^{Npq}\left|{V}_{i}-\:1\right|$$

- Improving voltage stability index.

The 4th technical OF aims to improve the Voltage Stability Index (VSI) by minimizing the L-index, which is described in Eqs. (10) to (12) as follows:

10 $$\:{\:Min\:F}_{4}\left(x,u\right)=Min\:\left(VSI\right)=Min\:\left(\text{max}\left({L}_{j}\right)\right),\:j=1,\:2,\:\dots\:\:{N}_{b}$$

11 $$\:{L}_{j}=\left|1-{\sum\:}_{i=1}^{{N}_{g}}{F}_{\text{ji}}\frac{{V}_{i}}{{V}_{j}}\angle\:\left({\theta\:}_{\text{ij}}+{\delta\:}_{i}-{\delta\:}_{j}\right)\right|$$

12 $$\:{F}_{\text{ji}}=-{\left[{Y}_{\text{LL}}\right]}^{-1}\left[{Y}_{\text{LG}}\right]$$

Where, F_ji_ is considered an element of complex matrix F that is given from the sub-matrices of admittance Y_LL_ and Y_LG_, N_g_ is number of generators, δ_i_ and δ_j_ are voltage phase angles of buses i and j. The matrices Y1 and Y2 are the system sub-matrices^44^.

#### Environmental category

The 5th OF is concerned with reducing emissions. The objective of minimizing the total emission level is pivotal for realizing environmental benefits within the power system. The total emission from generating units is calculated in ton/hour as in Eq. (13)^1^

13 $$\:{Min\:F}_{5}={\sum\:}_{i=1}^{Ng}1{0}^{-2}\left({\alpha\:}_{i}+{\beta\:}_{i}{P}_{{gi}}+{\gamma\:}_{i}\:{{P}_{gi}}^{2}\right)+\left|{\zeta\:}_{i}{exp}[{\lambda\:}_{i}Pgi]\right|\:\text{T}\text{o}\text{n}/\text{h}\text{r}$$

where, $\:{\alpha\:}_{i},{\beta\:}_{i},{\gamma\:}_{i},{\zeta\:}_{i},{\lambda\:}_{i}$ are the emission coefficients of generator i, $\:{P}_{gi}\:$represents the active power generated by the power unit situated at bus i, with Ng representing the total number of generators.

### Constraints

To achieve the optimal objectives most efficiently, it is essential to consider the operational constraints outlined in the active and reactive power flow as in Eqs. (14) and (15).

14 $$\:Q_{gi} - Q_{Li} + Q_{Ci} - V_{i} \sum \: _{{j = 1}}^{{N_{b} }} V_{j} \left( {G_{{{\text{ij}}}} {\text{sin}}\theta _{{{\text{ij}}}} - B_{{{\text{ij}}}} {\text{cos}}\theta _{{{\text{ij}}}} } \right) = 0,\:{\text{i = 1}},{\text{2}}, \cdots {\text{N}}_{{{\text{PQ}}}}$$

15 $$P_{{gi}} - P_{{Li}} - V_{i} \sum \: _{{j = 1}}^{{N_{b} }} V_{j} \left( {G_{{{\text{ij}}}} {\text{cos}}\:\theta _{{{\text{ij}}}} {\text{ + B}}_{{{\text{ij}}}} {\text{sin}}\:\theta _{{{\text{ij}}}} } \right) = 0,\:{\text{i = 1}},{\text{2}}, \cdots {\text{N}}_{{\text{b}}} {\text{ - slack}}$$

The inequality operational constraints outlined are maintained within the designated minimum and maximum bounds for each constraint, as indicated in Eqs. (16)– (18), ensuring the preservation of generator limitations. Equations (19)-(20) ensure the constrained operation of tapping points for transformers and shunt reactive power compensators. The voltage profile is kept within permissible operating limits, as specified in Eq. (21). Furthermore, Eq. (22) maintains the secure operation of transmission lines by constraining power flow within an acceptable range^46^.

16 $$\:{{\:\text{P}}_{{\text{g}}_{\text{i}}}}^{\text{m}\text{i}\text{n}}\le\:{\text{P}}_{{\text{g}}_{\text{i}}}\le\:{{\text{P}}_{{\text{g}}_{\text{i}}}}^{\text{m}\text{a}\text{x}\:}$$

17 $$\:{{\text{Q}}_{{\text{g}}_{\text{i}}}}^{\text{m}\text{i}\text{n}}\le\:{\text{Q}}_{{\text{g}}_{\text{i}}}\le\:{{\text{Q}}_{{\text{g}}_{\text{i}}}}^{\text{m}\text{a}\text{x}}$$

18 $$\:{{\:\text{V}}_{\text{g}\text{i}}}^{\text{m}\text{i}\text{n}}\le\:{\text{V}}_{\text{g}\text{i}}\le\:{{\text{V}}_{\text{g}\text{i}}}^{\text{m}\text{a}\text{x}}$$

19 $$\:{{\:\text{T}}_{\text{i}}}^{\text{m}\text{i}\text{n}}\le\:{\text{T}}_{\text{i}}\le\:{{\text{T}}_{\text{i}}}^{\text{m}\text{a}\text{x}}$$

20 $$\:{{\text{Q}}_{{\text{c}}_{\text{i}}}}^{\text{m}\text{i}\text{n}}\le\:{\text{Q}}_{{\text{c}}_{\text{i}}}\le\:{{\text{Q}}_{{\text{c}}_{\text{i}}}}^{\text{m}\text{a}\text{x}}$$

21 $$\:{{\text{V}}_{\text{L}\text{i}}}^{\text{m}\text{i}\text{n}}\le\:{\text{V}}_{\text{L}\text{i}}\le\:{{\text{V}}_{\text{L}\text{i}}}^{\text{m}\text{a}\text{x}}$$

22 $$\:{{\:\text{S}}_{\text{L}\text{i}}}^{\text{m}\text{i}\text{n}}\le\:{\text{S}}_{\text{L}\text{i}}\le\:{\text{S}}_{\text{L}\text{i}}^{\text{m}\text{a}\text{x}}$$

The collective *function F* in Eq. (23) consists of 5 individual OFs; *F*_1_, *F*_2_, *F*_3_, *F*_4_ and *F*_5,_ which can be formulated as follows:

23 $$\:F={\text{w}}_{1}.\:{\text{F}}_{1}+{\text{w}}_{2}.\:{\text{F}}_{2}+{\text{w}}_{3}.\:{\text{F}}_{3}+{\text{w}}_{4}.\:{\text{F}}_{4}+{\text{w}}_{5}.\:{\text{F}}_{5}$$

Where w_1_, w_2_, w_3_, w_4_, w_5_ are the weight factors where w_1_+w_2_+w_3_+w_4_+w_5_ equal 1.

## Proposed solution methodology

A recent algorithm ARO^47^, which was created from the survival strategies of rabbits in nature, as rabbits are herbivores that feed mainly on grass, forbs, and leafy weeds. There are two simulated strategies that have been devised detour eating and haphazard hiding. The initial approach, detour eating, seeks to deter predators from discovering rabbit nests by steering clear of grass consumption near their burrows. This tactic aligns with the well-known Chinese idiom: “rabbits do not eat the grass near their own nest.” This strategy is commonly referred to as exploration. Additionally, rabbits possess a broad field of vision, with a significant portion dedicated to scanning, enabling them to effortlessly locate food across expansive areas.

The alternative strategy is termed exploitation or random hiding. Rabbits consistently endeavor to reduce the risk of capture by potential threats. Their expertise in creating burrows allows them to elude hunters and predators. Rabbits construct multiple burrows in proximity to their nest, selecting one at random as a refuge from potential threats. However, there are instances where rabbits may experience energy depletion. Situated at the lower end of the food chain and facing numerous predators, rabbits must conserve energy for survival. This is achieved through switching between foraging and random hiding^48^. The mathematical model of the ARO described as follow^47^.

- *Detour foraging (exploration)*.

In this approach, rabbits are avoiding eating grass in close proximity to their burrows. Instead, they explore each other’s areas haphazardly in search of food. This behavior involves continual adjustments of their positions in relation to other rabbits within the group, introducing an element of disturbance. A model has been suggested to describe this strategic behavior.

24 $$\:{\overrightarrow{\:V}}_{i}\left(t+1\right)={\overrightarrow{\:x}}_{j}\:\left(t\right)+R.\:\left({\overrightarrow{\:x}}_{i}\:\left(t\right)-{\overrightarrow{\:x}}_{j}\:\left(t\right)\right)+round\left(0.5\:.\:\left(0.05+{r}_{1}\right)\right).{n}_{1},\:\:i,j=1,\dots\:..,n\:and\:j\ne\:i$$

25 $$\:R=L.c$$

26 $$\:L=(e-{e}^{{\left(\frac{t-1}{T}\right)}^{2}}.\:\text{s}\text{i}\text{n}(2\pi\:{r}_{2})$$

27 $$\:c\left(k\right)=\left\{\begin{array}{c}1\:\:\:\:\:\:\:\:\:\:\:\:\:\:\:\:\:\:\:\:if k=g\left(l\right)\\\:0\:\:\:\:\:\:\:\:\:\:\:\:\:\:\:\:\:\:\:\:\:\:\:\:\:\:\:\:\:\:\:\:\:\:\:else\end{array}\right.\:\:\text{k}=1,\dots\:\dots\:,\:\text{d}\:\text{a}\text{n}\text{d}\:\text{l}=\:1,\dots\:\dots\:.,\:[{r}_{3\:}.\:d]$$

28 $${\text{g}}={\text{ rand perm }}\left( {\text{d}} \right)$$

29 $$\:{n}_{1}\sim N\left(\text{0,1}\right)$$

where, the intrant position of the i^th^ rabbit at time t + 1 is denoted as $\:{\overrightarrow{\:V}}_{i}\left(t+1\right)$, $\:{\overrightarrow{\:x}}_{j}\left(t\right)$represents the position of the i^th^ rabbit at time t. In this context, n refers to the size of the rabbit population, while d indicates the dimension of the problem. T represents the maximum number of iterations. The function [$\:.\:]$ corresponds to the ceiling integer function. The function rand perm generates a random permutation of integers ranging from 1 to d. Additionally, r_1_, r_2_, and r_3_ are three random numbers belong to (0,1). The running length, denoted as l, signifies the movement pace during the detour foraging process. Lastly, n1 subject to the standard normal distribution. Equation (24) demonstrates that search individuals engage in a random exploration based on the positions of other individuals. This behavior allows a rabbit to move far from its own territory and venture into the territories of other rabbits. Notably, when a rabbit visits the nests of others instead of its own nest, it makes a significant contribution to the exploration process and enhances the ability of the ARO algorithm to detect global search.

During each iteration in the ARO algorithm, the rabbit generates multiple burrows (d burrows) in a randomized manner around its current position across each dimension of the search space. The purpose of creating these burrows is to provide hiding options for the rabbit, reducing the likelihood of being targeted or attacked. The specific j^th^ burrow for the i^th^ rabbit is generated by:

30 $$\:{\overrightarrow{\:b}}_{i,j}\left(t\right)={\overrightarrow{\:x}}_{j}\:\left(t\right)+H\:.\:g.\:{\overrightarrow{\:x}}_{i}\:\left(t\right),\:\:i=1,\dots\:..,n\:and\:j=1,....,d$$

31 $$\:H=\:\frac{T-t+1}{T}\:.\:{r}_{4}$$

32 $$\:{n}_{2}\:\sim\:N\left(\text{0,1}\right)$$

33 $$\:g\left(k\right)=\left\{\begin{array}{c}1\:\:\:\:\:\:\:\:\:\:\:\:\:\:\:\:\:\:\:\:if k=j\\\:\:\:0\:\:\:\:\:\:\:\:\:\:\:\:\:\:\:\:\:\:\:\:\:\:\:\:\:\:else\:\:\:\:\:\:\:\:\:\end{array}\right.\:\:\text{k}=1,\:\dots\:\dots\:,\:\text{d}\:\:\:\:\:\:\:\:\:\:$$

Based on Eq. (30), the d burrows are generated within the vicinity of a rabbit along each dimension. The hiding parameter H plays a crucial role, gradually decreasing linearly from 1 to 1/T throughout the iterations, with the addition of random perturbations. This parameter determines the size of the neighborhood in which the burrows are initially created. Initially, a larger neighborhood is considered, but as the iterations progress, this neighborhood gradually decreases in size.

When seeking shelter, rabbits employ a random selection strategy from their available burrows. To mathematically represent this random hiding behavior, Eqs. (34)-(36) are employed:

34 $$\:{\overrightarrow{\:V}}_{i}\left(t+1\right)={\overrightarrow{\:x}}_{i}\:\left(t\right)+R.\:\left({r}_{4}\:{\overrightarrow{.\:b}}_{i,r}\left(t\right)-{\overrightarrow{\:x}}_{i}\:\left(t\right)\right),\:\:i=1,\dots\:..,n$$

35 $$\:g\left(k\right)=\left\{\begin{array}{c}1\:\:\:\:\:\:\:\:\:\:\:\:\:\:\:\:\:\:\:\:if k=[{r}_{5\:}.\:d]\\\:\:\:0\:\:\:\:\:\:\:\:\:\:\:\:\:\:\:\:\:\:\:\:\:\:\:\:\:\:else\:\:\:\:\:\:\:\:\:\end{array}\right.\:\text{k}=1,\:\dots\:\dots\:,\:\text{d}\:$$

36 $$\:{\overrightarrow{\:b}}_{i,r}\left(t\right)={\overrightarrow{\:x}}_{j}\:\left(t\right)+H\:.\:{g}_{r}.\:{\overrightarrow{\:x}}_{i}\:\left(t\right)$$

In the given equation, $\:{\overrightarrow{\:b}}_{i,r}\left(t\right)$represents a randomly chosen burrow for hiding from the set of d burrows available to the rabbit. The variables r_4_ and r_5_ represent two random numbers within the range of (0, 1).

Referring to Eq. (34), the i^th^ search individual aims to update its position towards the selected burrow from the d burrows. Once either the detour foraging or random hiding is successfully executed, the position of the i^th^ rabbit is updated according to the following expression:

37 $$\:{\overrightarrow{\:x}}_{i}\left(t+1\right)=\left\{\begin{array}{c}{\overrightarrow{\:x}}_{i}\left(t\right)\:\:\:\:\:\:\:\:f\:\left(\:{\overrightarrow{\:x}}_{i}\left(t\right)\right)\:\:\le\:\:\:f\:\left(\:{\overrightarrow{\:v}}_{i}\left(t+1\right)\right)\:\:\:\:\:\:\:\:\:\:\:\\\:{\overrightarrow{\:v}}_{i}\left(t+1\right)\:\:\:\:\:f\:\left(\:{\overrightarrow{\:x}}_{i}\left(t\right)\right)\:\:>\:\:f\:\left(\:{\overrightarrow{\:v}}_{i}\left(t+1\right)\right)\:\:\:\:\:\end{array}\right.$$

(b) *Exploration to exploitation*.

The switching from exploration to exploitation is carried by the energy factor of the proposed ARO as:

38 $$\:A\left(t\right)=4(1-\frac{t}{T})\:ln\frac{1}{r}$$

where “r” represents a randomly generated number that falls in the range between 0 and 1.

**Fig. 1:**
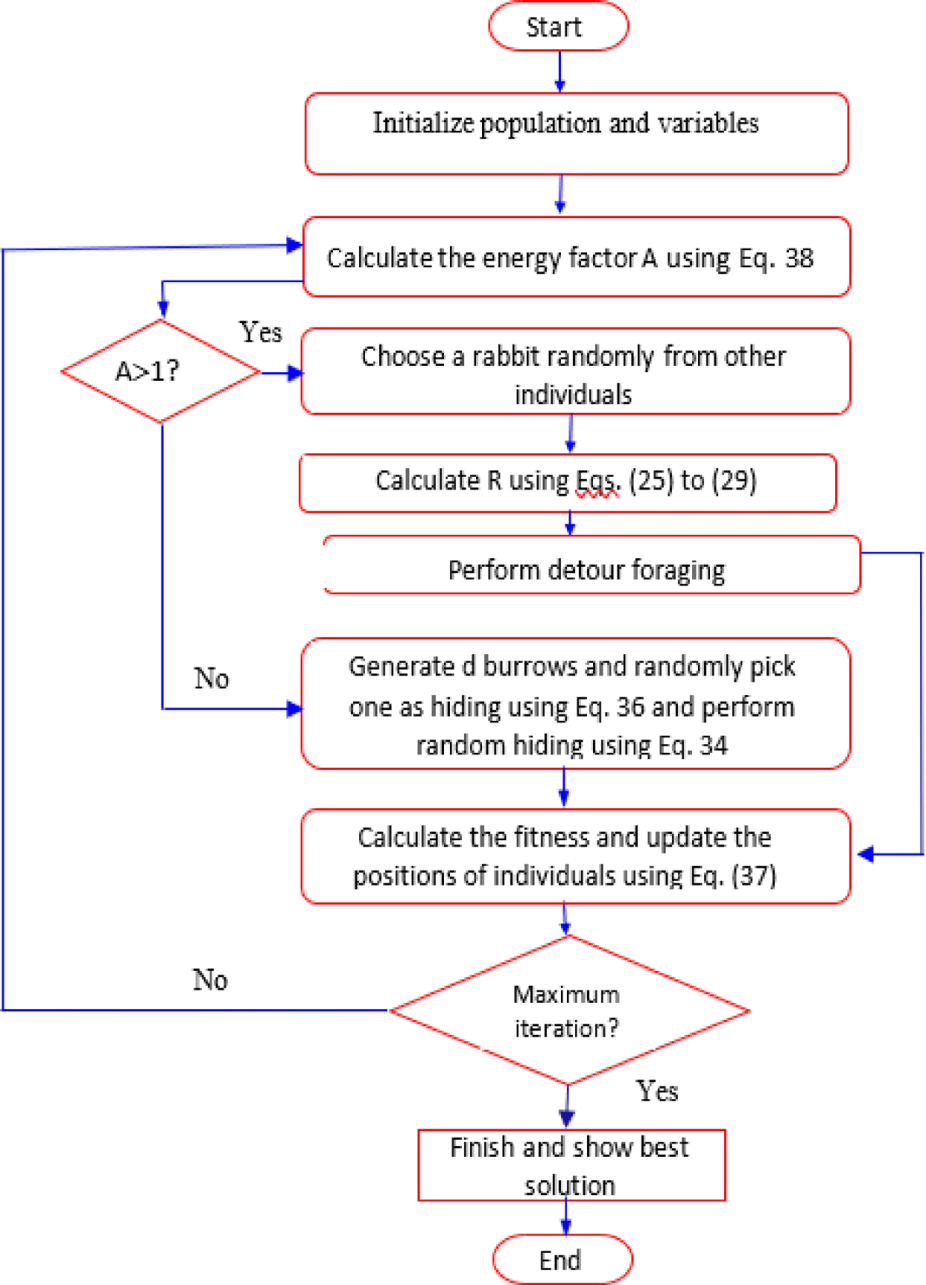
Flowchart of ARO.

The ARO behavior of rabbits varies depending on the energy factor A(t). When A(t) is greater than 1, rabbits are inclined to explore different foraging areas randomly during the exploration phase. This is referred to as “detour foraging.” Conversely, when A(t) is less than or equal to 1, rabbits are motivated to exploit their burrows randomly during the exploitation phase, resulting in “random hiding.” As the number of iterations increases, A gradually decreases, which enables individuals within the rabbit population to alternate between detour foraging and random hiding behaviors. These updates efficiently continue until the termination criterion is met, at which point the best solution is identified and returned^48^. Further details of the flow chart of ARO algorithm are shown in Fig. 1.

## Experimental simulations

### Test systems

Experiments were conducted using six IEEE standard power systems: the IEEE 30-bus system and the IEEE 57-bus system are considered two small size systems and IEEE 300, 1354, 3012, and 9241 as large test systems. In the IEEE 30-bus system, there were 6 generation buses, 21 loads, 41 branches, 4 tap changers, and 3 shunt capacitors. On the other hand, the IEEE 57-bus system consisted of 7 generation buses, 80 branches, 17 tap changers, and 3 shunt capacitors. The other four systems are considered large-scale systems, are chosen to test the proposed algorithm and assess the performance of it. For the proposed ARO algorithm, the population size was set to 100, and the maximum number of iterations was set to 200 with the IEEE-30 bus system and 300 with IEEE-57 bus system. The dimensions of the problem were determined based on the specific tested power system being considered.

### Defination of studied cases

Table 2 reports the classification of the studied cases. These cases were classified into different categories based on the following criteria: technical, economic, and environmental. By organizing the cases into these categories, a comprehensive analysis was carried out to assess the performance of the different objective functions and evaluate the benefits achieved in terms of technical, economic, and environmental aspects. The ARO algorithm was tested on both single and multi OFs for the OPF problem. Additionally, these objective functions were combined in double, triple, and multi-objective formulations. The algorithm’s effectiveness was evaluated based on its ability to optimize the aforementioned objectives in these complex systems.

**Table 2:** Definition of the OPF cases studied of single and multi-OF frameworks.

Test system/s control variables	Case #	Number of considered Objectives	Objectives Aspects				
			Economical	Technical			Environmental
			Cost minimization	VD	VS	PL	Emission
			OF1	OF3	OF5	OF2	OF4
IEEE 30/ 25	1	1	√				
	2	1		√			
	3	1			√		
	4	1				√	
	5	1					√
	6	2	√				√
	7	2	√			√	
	8	2	√		√		
	9	2	√	√			
	10	3	√	√		√	
	11	3	√			√	√
	12	3	√	√			√
	13	4	√	√		√	√
	14	5	√	√	√	√	√
IEEE 57/ 34	15	1	√				
	16	1				√	
	17	2	√			√	
	18	3	√	√		√	
IEEE-300-bus/ 259	19	1	√				
IEEE-1354-bus/ 1836	20	1	√				
IEEE 3012 bus/ 1214	21	1	√				
IEEE 9241 bus/ 11536	22	1	√				

## Numerical simulation applications

This section reports the numerical simulation results for the defined cases studied in Table 2. There are 22 cases that were analyzed, as outlined in Table 2, encompassing two small-sized test systems and four large-scale test systems spanning a range of 300 to 9241 buses. The performance of ARO was assessed against recent published algorithms. The simulation was carried out on a Core I7 laptop with 8 GB of RAM. The proposed ARO was implemented and evaluated using MATLAB R2020a.

### The first test system

The analysis focuses on the first test system, IEEE 30-bus, investigating 14 different cases that encompass up to 5 OFs. For cases 1–5, Tables 3 and 4 outline respectively the values of single OF cases and the associated settings of the system control variables. In Case 1, the primary OF1 is minimizing the fuel cost. The simulation analysis shows that the fuel cost achieved is reported as $798.943 per hour. For Case 2, the focus shifts towards decreasing the load-buses voltage deviation. The simulation results reveal that by employing the ARO algorithm, a voltage deviation (VD) of 0.093 per unit is obtained. In Case 3, the voltage stability index is reported as 0.109 p.u. This index provides an indication of the system’s voltage stability. Case 4 achieves the lowest power loss of 2.881 MW. This means that the system operates with minimal power dissipation. Furthermore, Case 5 demonstrates the lowest emission rate observed, which amounts to 0.2047 tons/kg. This indicates a more environmentally friendly operation.

**Fig. 2:**
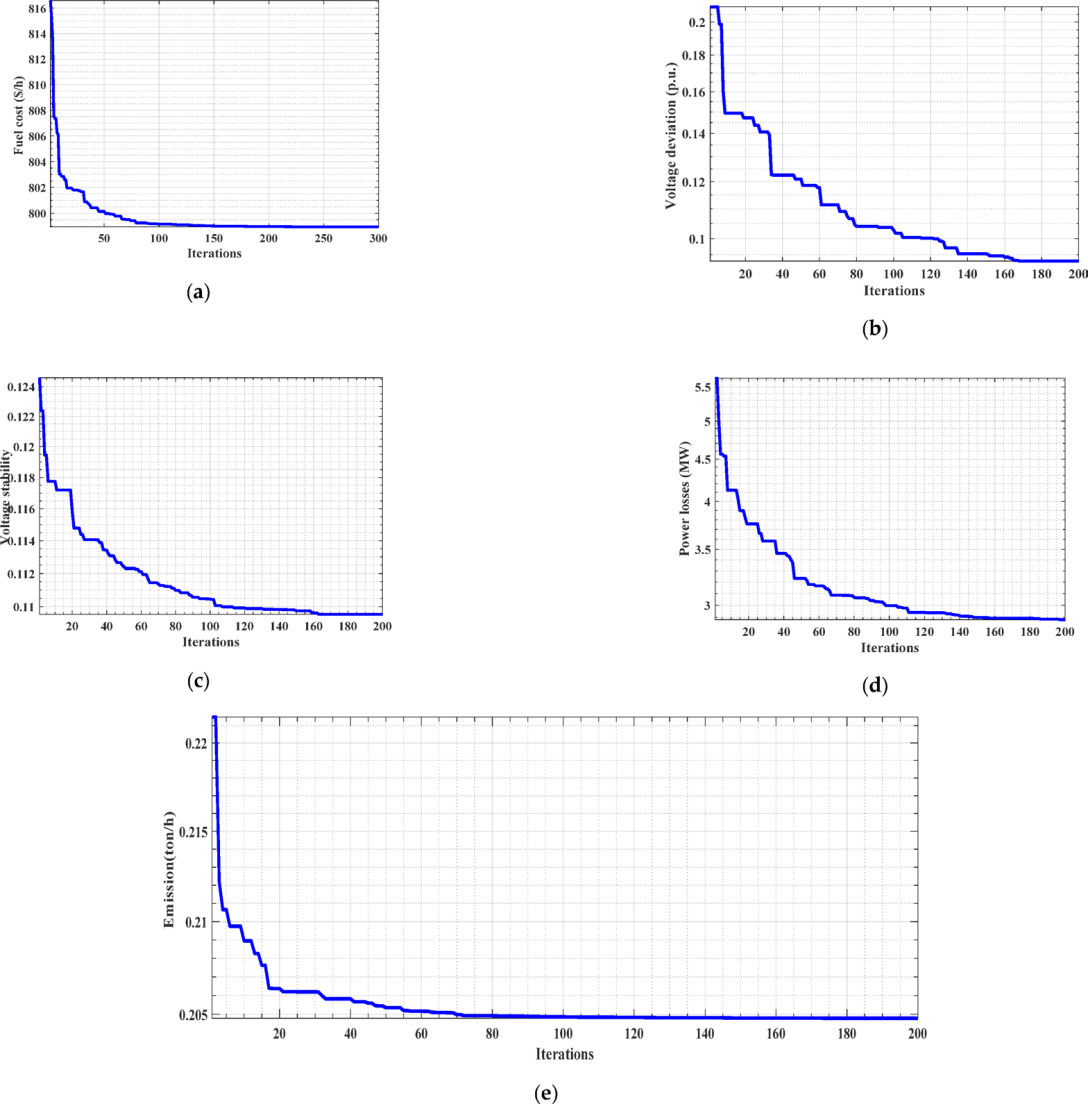
The convergence curves for the single objective cases (Cases 1–5). (**a**) fuel cost (Case 1), (**b**) voltage deviation (Case 2), (**c**) Voltage stability (Case 3), (**d**) Power losses (Case 4), (**e**) Emission minimization (Case 5).

Figures 2 exhibit favorable convergence characteristics when utilizing the ARO algorithm in all investigated cases for Cases 1–5. These figures illustrate the convergence behavior of the ARO algorithm. The convergence curves clearly indicate that the ARO algorithm rapidly converges to the optimal solution and maintains stability thereafter. Table 5 compares the ARO algorithm and recent optimizers that were customized from the literature. Also, the proposed ARO algorithm consistently delivers the most competitive solutions for various objectives. This comparison highlights the superiority of the ARO algorithm in optimizing the system’s performance.

**Table 3:** Single OFs of the first test system using ARO (Cases 1–5).

Objective functions	Case 1	Case 2	Case 3	Case 4	Case 5
Fuel cost ($/h) (OF1)	798.943	831.27	835.61	963.79	943.92
VD (p.u.) (OF3)	1.885	0.093	3.343	2.090	1.447
VS (OF5)	0.1269	0.148	0.109	0.125	0.1328
PL (MW) (OF2)	8.612	8.514	5.77	2.881	3.069
Emission (ton/h) (OF4)	0.366	0.275	0.250	0.2068	0.2047

**Table 4:** Control variables of single objective functions for IEEE 30-bus test system using ARO.

VARs	Min.	Max.	Case 1	Case 2	Case 3	Case 4	Case 5
PG_1_ (MW)	50	200	177.31	131.083	117.666	52.829	63.920
PG_2_ (MW)	20	80	48.700	73.283	62.565	78.786	67.571
PG_5_ (MW)	15	50	21.059	23.551	33.684	49.981	49.996
PG_8_ (MW)	10	35	20.972	24.503	31.914	34.919	34.987
PG_11_ (MW)	10	30	11.943	25.411	22.742	29.858	29.998
PG_13_ (MW)	12	40	12.025	14.084	20.599	39.908	39.996
V_1_ (p.u.)	0.95	1.1	1.100	0.995	1.100	1.100	1.100
V_2_ (p.u.)			1.087	1.000	1.097	1.096	1.092
V_5_ (p.u.)			1.059	1.018	1.100	1.076	1.076
V_8_ (p.u.)			1.067	1.007	1.100	1.086	1.076
V_11_ (p.u.)			1.099	1.012	1.099	1.092	1.079
V_13_ (p.u.)			1.100	1.046	1.098	1.099	1.089
QC_10_ (Mvar)	0	5	4.637	4.592	4.966	4.112	2.526
QC_12_ (Mvar)			4.976	3.170	4.453	4.409	1.036
QC_15_ (Mvar)			3.929	4.811	4.733	4.632	4.494
QC_17_ (Mvar)			4.838	1.770	4.778	4.757	4.160
QC_20_ (Mvar)			4.133	4.726	4.788	2.112	4.580
QC_21_ (Mvar)			4.814	4.121	4.716	3.917	2.228
QC_23_ (Mvar)			3.396	4.894	4.867	4.530	3.174
QC_24_ (Mvar)			4.985	4.817	4.983	3.894	3.626
QC_29_ (Mvar)			2.506	1.892	4.927	2.941	1.949
T6-9	0.9	1.1	1.020	1.028	0.903	0.984	1.014
T6-10			0.924	0.903	0.905	0.955	0.966
T4-12			0.987	1.049	0.901	0.983	1.029
T28-27			0.963	0.961	0.900	0.969	0.990

**Table 5:** Comparison between ARO & recent optimization algorithms for single ofs, (Cases 1–5).

Case	ARO	SSO^29^	PSO^29^	DA-PSO^49^	DA-APSO^49^	ECHT^24^	MVO^50^	WOA-PS^51^
1	**798.943**	799.41	801.23	802.12	802.63	800.41	799.24	799.56
2	**0.093**	1.54	1.61	-	-	-	-	-
3	**0.109**	0.125	0.125	-	-	0.136	0.115	-
4	**2.881**	2.902	3.278	3.189	3.003	3.084	2.881	2.967
5	**0.2047**	0.205	0.205	0.205	-	0.205	-	0.206

**Fig. 3:**
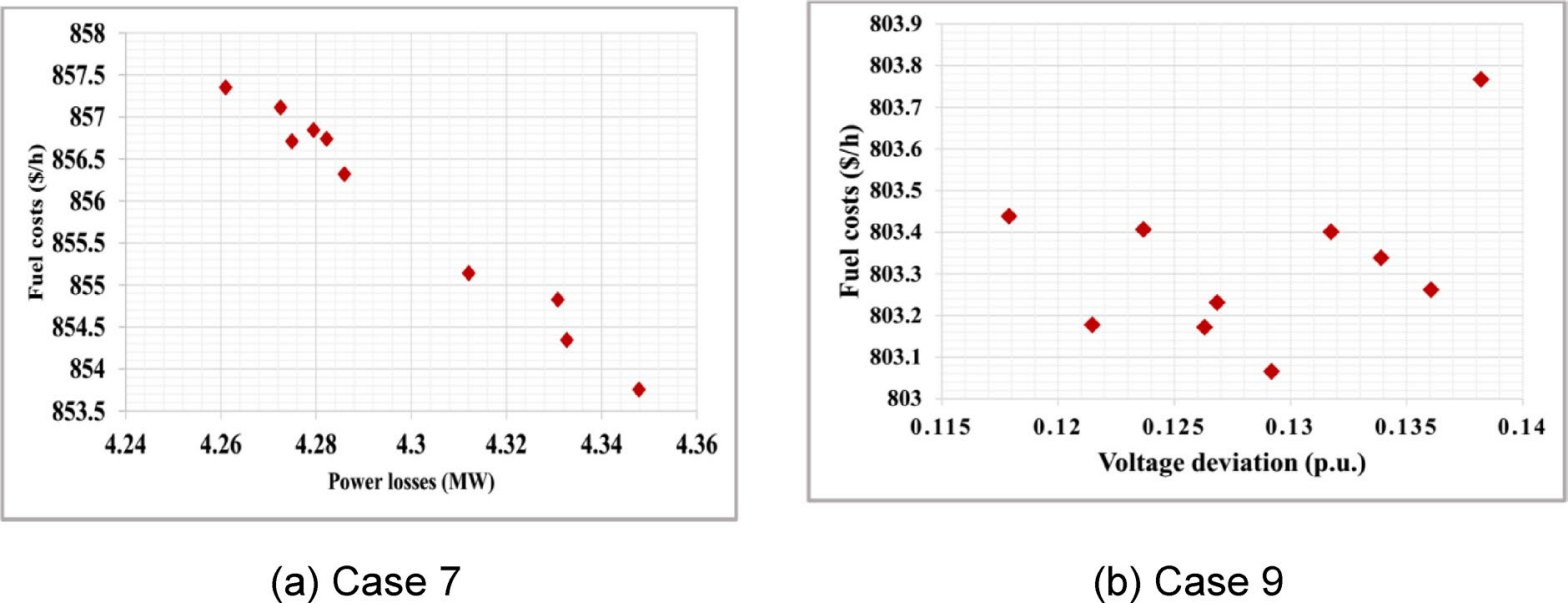
Bi-objective Pareto set of OPF: (**a**) Fuel costs and power losses (Case 7); (**b**) Fuel costs and voltage deviation (Case 9).

To consider multiobjective functions including bi-, tri- and four objective functions, Table 6 reports the results for Cases 6–14. The table provides an overview of the outcomes achieved through the optimization process for each case, considering multiple objectives. The results reported in Table 6 constitute objectives followed by the control variables of the tested cases. The simulation results using the proposed ARO algorithm are compared to other related works in the literature^23,49,52–55^. Table 7 shows that the proposed ARO leads to the most efficient solutions for the considered Cases 6–9. Figure 3 shows Pareto sets for Cases 7 and 9. Cases 10–12 address three objectives functions by the proposed ARO algorithm. The results are tabulated in Table 8. In Case 10, three objectives, fuel costs, voltage deviation, and power losses are carried out. Case 11 focuses on minimizing fuel costs, power losses, and emission levels, while Case 12 optimizes fuel costs, voltage deviation, and emission levels simultaneously. The effectiveness of the proposed ARO algorithm is validated compared with the reported results in^23,52,53,55,56^. Table 9 clearly demonstrates that the proposed ARO algorithm leads to improved fuel costs and power losses compared to PSO-SSO^52^ in Case 10.

**Fig. 4:**
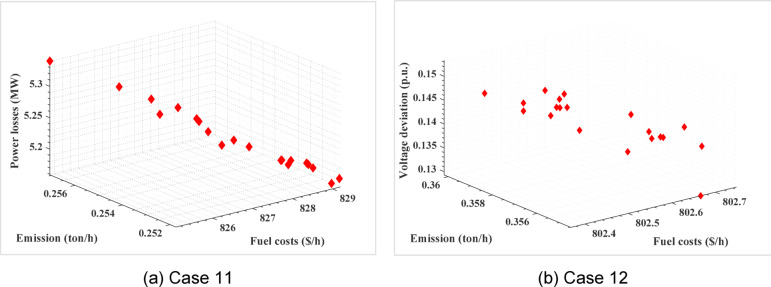
Tri-objective Pareto set of OPF in the first test system: (**a**) Fuel costs, power losses, and emission (Case 11); (**b**) Fuel costs, Voltage deviation, and emission (Case 12).

Furthermore, in Cases 11–12, the economic, technical, and environmental benefits surpass those achieved by PSO, SSO, PSO-SSO^52^, and MOAD^53^. The Pareto solutions for Cases 10–12 are illustrated in Fig. 4. The simulation results obtained by the ARO and are compared with other methods, Objectives ECHIT^24^, PSO^29^, PSO-SSO^29^, SSO^29^, I-NSGA-III^42^, MODA^57^ and Jaya^56^, is presented in Table 9. In Case 13, the optimization is performed simultaneously for fuel costs, voltage deviation, power losses, and emission levels. Case 14 involves considering five objective functions. In both cases, Acceptable economic, technical, and environmental benefits are achieved compared to the competitive algorithms PSO-SSO and MODA. These comparative studies validate the effectiveness and capability of the proposed ARO algorithm.

**Table 6:** OPF solution and control variables for IEEE-30 bus system using the ARO algorithm for cases 6–14.

VARs	Case 6	Case 7	Case 8	Case 9	Case 10	Case 11	Case 12	Case 13	Case 14
Fuel cost ($/h)	**800.8**	**854.1**	**799.43**	803.43	**826.61**	**827.5**	**802.66**	**826.29**	**826.84**
VD (p.u.)	1.693	1.837	2.462	**0.1178**	**0.448**	1.838	**0.129**	**0.461**	**0.471**
VS	0.129	0.127	**0.117**	0.1487	0.146	0.128	0.148	0.146	**0.144**
PL (MW)	7.654	**4.345**	8.699	9.845	**5.488**	**5.218**	9.309	**5.486**	**5.461**
Emission (ton/h)	**0.328**	0.231	0.365	0.3684	0.257	**0.254**	**0.354**	**0.256**	**0.256**
PG_1_ (MW)	162.43	105.02	178.19	176.98	124.77	122.97	172.85	124.69	124.36
PG_2_ (MW)	50.886	54.254	47.514	48.186	52.591	51.975	49.973	52.507	52.865
PG_5_ (MW)	22.676	37.233	21.098	21.142	31.566	30.863	21.601	30.880	31.474
PG_8_ (MW)	26.567	34.918	20.333	21.475	34.853	34.943	23.054	34.872	34.934
PG_11_ (MW)	14.309	29.903	13.872	12.228	24.828	26.164	12.656	25.579	25.299
PG_13_ (MW)	14.180	26.416	12.230	12.086	20.276	21.697	12.571	20.351	19.924
V_1_ (p.u.)	1.100	1.100	1.042	1.100	1.099	1.100	1.053	1.100	1.100
V_2_ (p.u.)	1.086	1.093	1.026	1.087	1.084	1.091	1.031	1.088	1.088
V_5_ (p.u.)	1.060	1.074	1.012	1.059	1.056	1.068	1.003	1.061	1.060
V_8_ (p.u.)	1.068	1.079	1.004	1.066	1.067	1.080	1.004	1.069	1.071
V_11_ (p.u.)	1.095	1.097	1.013	1.097	1.032	1.096	1.041	1.045	1.045
V_13_ (p.u.)	1.099	1.096	1.014	1.099	1.034	1.098	1.012	1.015	1.026
QC_10_ (Mvar)	4.525	4.019	2.879	4.867	2.336	4.430	3.383	4.524	2.191
QC_12_ (Mvar)	2.735	2.482	1.757	3.456	1.953	4.311	0.963	2.958	2.852
QC_15_ (Mvar)	4.385	3.988	3.941	4.043	3.898	4.408	4.296	4.421	2.927
QC_17_ (Mvar)	4.428	3.247	2.811	4.535	4.159	3.789	1.914	3.603	3.295
QC_20_ (Mvar)	3.362	3.535	4.941	4.964	4.648	3.902	4.942	3.892	4.810
QC_21_ (Mvar)	4.135	4.710	2.892	4.058	3.848	4.778	4.725	4.632	3.915
QC_23_ (Mvar)	3.580	2.109	4.754	4.778	4.975	2.948	4.355	2.581	3.342
QC_24_ (Mvar)	4.138	4.663	4.622	4.666	2.391	4.861	4.845	4.552	4.709
QC_29_ (Mvar)	1.897	2.925	2.926	2.839	2.336	2.166	1.748	2.501	1.811
T6-9	1.002	1.001	1.016	1.100	1.094	1.021	1.059	1.097	1.090
T6-10	0.959	0.965	0.904	0.976	0.975	0.943	0.910	1.011	1.002
T4-12	1.003	1.001	0.985	0.909	1.073	1.003	0.985	1.040	1.068
T28-27	0.969	0.975	0.969	0.935	1.029	0.978	0.965	1.032	1.018

**Table 7:** ARO and recent optimizers from the literature for multi-OFs for (Cases 6–9).

Case	Objective	MSA^54^	PSO^29^	EMSA^54^	MODA^57^	DA-APSO ^49^	MOFA-CPA ^23^	PSO-SSO [29]	ECHIT^24^	ARO
6	OF1	834.1532	834.95	8.33.977	8.38.604	-	852.02	834.80	**-**	**800.8**
	OF4	0.3286	0.243	0.3293	0.254	-	0.279	0.243	**-**	**0.328**
7	OF1	856.2673	-	859.9514	-	-	-	-	-	**854.1**
	OF2	9.9012	-	4.9012	-	-	-	-	-	**4.345**
8	OF1	800.0275	834.4	799.3582	-	-	-	830.35	**-**	**799.43**
	OF5	0.1209	0.128	0.1209	-	-	-	0.125	**-**	**0.117**
9	OF1	803.8740	804.48	803.4286	807.2807	802.63	-	803.99	803.72	**803.43**
	OF3	0.1180	0.126	803.8740	0.023	0.116	-	0.094	0.095	**0.1178**

**Table 8:** Results obtained by ARO and other optimizers for tri-OFs.

Case #	OF #	PSO^29^	MODA^53^	SSO^29^	Jaya^56^	MOFA-CPA^23^	PSO-SSO^29^	ARO
10	OF1	889.58	-	858.88	826.44	-	864.27	**826.61**
	OF3	0.353	-	0.353	0.2662	-	0.316	**0.448**
	OF2	4.712	-	4.712	6.611	-	4.545	**5.488**
11	OF1	864.584	867.907	867.034	858.9	878.13	865.18	**827.5**
	OF2	4.197	4.5342	4.148	4.622	3.9232	4.093	**5.218**
	OF3	0.225	0.2640	0.223	0.233	0.2165	0.224	**0.254**
12	OF1	814.833	-	807.94	834.06	-	804.332	**802.66**
	OF3	0.156	-	0.166	0.1989	-	0.164	**0.129**
	OF4	0.343	-	0.313	0.2511	-	0.346	**0.354**

**Table 9:** ARO against recent optimizers for cases 13 and 14 for the IEEE 30-bus system.

Case	Objectives	ECHIT^24^	PSO^29^	PSO-SSO^29^	SSO^29^	I-NSGA-III^42^	MODA^57^	Jaya^56^	Proposed ARO
13	OF1	803.210	828.290	826.940	829.978	881.940	828.490	-	826.290
	OF3	0.296	0.550	0.466	0.516	0.175	0.585	-	0.461
	OF2	5.586	5.644	5.515	5.426	4.745	5.912	-	5.486
	OF4	0.253	0.261	0.258	0.250	0.221	0.265	-	0.256
14	OF1		828.290	826.800	827.780	843.857	-	812.180	826.840
	OF3		0.550	0.463	0.550	0.239	-	0.191	0.471
	OF5		0.250	0.145	0.145	0.125	-	0.134	0.144
	OF2		5.644	5.464	5.644	5.741	-	9.003	5.461
	OF4		0.261	0.256	0.261	0.149	-	0.316	0.256

### Simulation results of IEEE 57-bus system

The 2nd test system, IEEE 57-bus system, has seven generation buses and 80 branches. The data is reported in^59^. To evaluate the capability of the ARO algorithm in handling single and multi-objective functions (OFs). In Cases 15 and 16, a single objective function was applied to minimize fuel costs (OF1) and power losses (OF2), respectively. Case 17 involved bi-objectives, aiming to optimize both OF1 and OF2 simultaneously. Lastly, in Case 18, three objectives, OF1, OF2, and OF3 are optimized simultaneously. Cases 15–18 present three algorithms called PSO, MFO and the proposed ARO. It was proven that the ARO has high ability, efficiency, and effectiveness compared with other competitive algorithms.

Table 10 presents the simulation results of the OPF problem for Cases 15–18, comparing the proposed ARO algorithm with two other competitive algorithms. In Case 15, the ARO algorithm achieved the lowest fuel costs of $41,672.88 per hour, while the PSO and MFO algorithms resulted in fuel costs of $41,727.01 and 41,695.26 $ per hour, respectively. Thus, it is evident that the ARO algorithm yields the minimum fuel costs. For Case 16, the proposed ARO algorithm reported an active power loss of 9.076 MW. In Case 17, the ARO algorithm produced the best results with fuel costs of $41,689.29 per hour and an active power loss of 13.89 MW. Finally, in Case 18, the optimal results were obtained using the ARO algorithm, resulting in fuel costs of $41,703.31 per hour, an active power loss of 14.356 MW, and a voltage deviation of 0.967 p.u. The settings of control variables for ARO against recent optimization algorithms applied on IEEE-57 bus test system for Cases 15–18 is shown in Table 11.

**Table 10:** Simulation results obtained by ARO and recent optimization methods for cases 15–18.

Case	Case 15			Case 16			Case 17			Case 18		
Algorithm	ARO	PSO	MFO	ARO	PSO	MFO	ARO	PSO	MFO	ARO	PSO	MFO
OF1 ($/h)	**41**,**672.88**	**41**,**727.01**	**41**,**695.265**	46,020.05	45,964.54	**49**,**360.1**	41,689.29	**41**,**905.8**	**41**,**792.5**	**41**,**703.31**	**42**,**031.22**	**42**,**010.03**
OF4 (p.u.)	1.611	1.594	1.545	4.903	2.57	-	1.57	-	**-**	**0.967**	**1.579**	**1.392**
*OF3 (ton/h)*	1.372	*1.39046*	*1.364*	*1.171*	*1.162*	*0.94795*	*1.348*	*1.3489*	*1.304*	1.325	*1.516*	*1.329*
OF2 (MW)	15.22	16.159	15.266	**9.076**	**10.480**	**11.295**	**13.89**	**13.715**	**13.914**	**14.356**	**16.949**	**15.713**
CPU time(sec)	**174.9**	**168.34**	**167.2**	174.38	191.76	180.75	171.6	**165.4**	**166.8**	**168.2**	**166.5**	**165.93**

**Table 11:** The settings of control variables for ARO against recent optimization methods for cases 15–18.

Case	Case 15			Case 16				Case 17			Case 18		
Algorithm	ARO	PSO	MFO	ARO	PSO	MFO		ARO	PSO	MFO	ARO	PSO	MFO
PG1 (MW)	144.64	139.884	143.224	191.141	199.01	193.411		145.81	134.996	143.32	144.054	145.81	142.5
PG2 (MW)	88.402	97.560	100.000	14.486	2.67	100.000		86.666	100.000	85.776	86.656	100.00	74.13
PG3 (MW)	46.286	45.855	43.563	123.836	140.00	140.000		46.232	43.791	45.996	46.030	46.56	45.15
PG6 (MW)	73.559	69.763	62.055	91.567	100.00	100.000		60.445	55.990	74.821	72.111	100.00	100.00
PG8 (MW)	467.354	456.203	462.889	330.678	309.59	273.605		449.918	433.882	439.969	444.321	466.99	421.93
PG9 (MW)	87.025	100.000	92.918	98.799	100.00	100.000		98.841	85.663	100.000	96.559	0.00	72.80
PG12 (MW)	358.749	358.532	361.417	409.368	410.00	355.555		376.786	410.000	371.100	375.426	410.00	410.000
V_1_ (p.u)	1.0660	1.0560	1.0600	1.0890	1.100	1.1000		1.0700	1.0720	1.093	1.0470	1.040	1.080
V_2_ (p.u)	1.0630	1.0540	1.0570	1.0880	1.100	1.1000		1.0660	1.0710	1.090	1.0440	1.040	1.070
V_3_ (p.u)	1.0560	1.0440	1.0510	1.0840	1.100	1.1000		1.0570	1.0620	1.083	1.0360	1.040	1.060
V_6_ (p.u)	1.0620	1.0530	1.0660	1.0890	1.100	1.0960		1.0650	1.0700	1.100	1.0510	1.070	1.060
V_8_ (p.u)	1.0720	1.0550	1.0870	1.0980	1.100	1.100		1.0810	1.0950	1.100	1.0660	1.100	1.070
V_9_ (p.u)	1.0470	1.0310	1.0600	1.0880	1.100	1.1000		1.0570	1.0760	1.074	1.0350	1.050	1.040
V_12_ (p.u)	1.0520	1.0290	1.0620	1.0780	1.100	1.0900		1.0610	1.1000	1.069	1.0300	1.030	1.040
Qc18 (Mvar)	9.229	20.000	20.000	7.944	0.000	20.000		12.6660	0.0000	0.000	6.828	20.000	4.990
Qc25 (Mvar)	13.069	10.581	19.677	12.303	18.68	12.823		14.867	13.344	11.280	11.577	0.00	20.00
Qc53 (Mvar)	12.998	19.105	13.761	12.711	20.00	9.263		10.532	0.000	20.000	12.699	0.00	12.47
T4-18	1.061	0.900	0.951	0.917	0.90	0.929		0.982	0.900	1.100	0.964	1.09	0.90
T4-18	0.984	1.100	1.097	1.058	0.90	1.100		1.028	1.100	0.939	1.044	1.04	1.10
T21-20	1.044	1.100	1.034	1.069	0.90	1.100		0.991	1.100	1.033	0.993	1.10	1.00
T24-25	1.040	0.900	1.072	1.002	0.90	1.100		0.993	1.066	1.100	1.010	1.01	1.01
T24-25	0.981	1.100	1.100	1.039	1.10	0.900		1.034	1.003	0.900	0.968	0.94	1.10
T24-26	1.029	1.063	1.040	1.028	1.00	0.989		1.005	1.029	1.064	1.028	1.04	1.02
T7-29	0.999	1.034	1.012	0.945	1.10	0.900		1.002	1.008	1.072	1.010	1.02	1.02
T34-32	0.946	0.900	0.997	0.955	0.90	0.934		0.957	0.900	0.943	0.943	0.90	0.90
T11-41	0.922	0.956	0.901	0.939	0.97	0.900		0.943	0.914	0.900	0.918	0.90	1.10
T15-45	0.979	0.966	0.977	0.942	1.10	0.900		0.979	0.998	1.000	0.969	0.90	0.99
T14-46	0.967	0.954	0.973	0.935	1.10	0.900		0.987	0.991	0.989	0.965	0.96	0.99
T10-51	0.981	0.962	0.993	0.942	1.10	0.910		0.995	1.013	0.995	0.979	0.98	0.90
T13-49	0.941	0.927	0.956	0.906	1.04	0.900		0.955	0.965	0.962	0.934	0.90	1.10
T11-43	0.971	0.957	1.100	0.961	1.10	0.900		0.977	1.100	1.036	0.965	1.03	0.90
T40-56	1.000	0.900	1.100	1.045	1.10	1.006		0.986	1.100	1.100	0.969	1.10	1.10
T39-57	0.964	0.900	0.900	1.025	0.90	0.984		0.973	0.993	0.900	0.977	0.93	0.90
T9-55	0.991	1.100	1.021	0.948	1.10	0.906		1.004	1.015	1.100	1.014	1.10	1.02

**Fig. 5:**
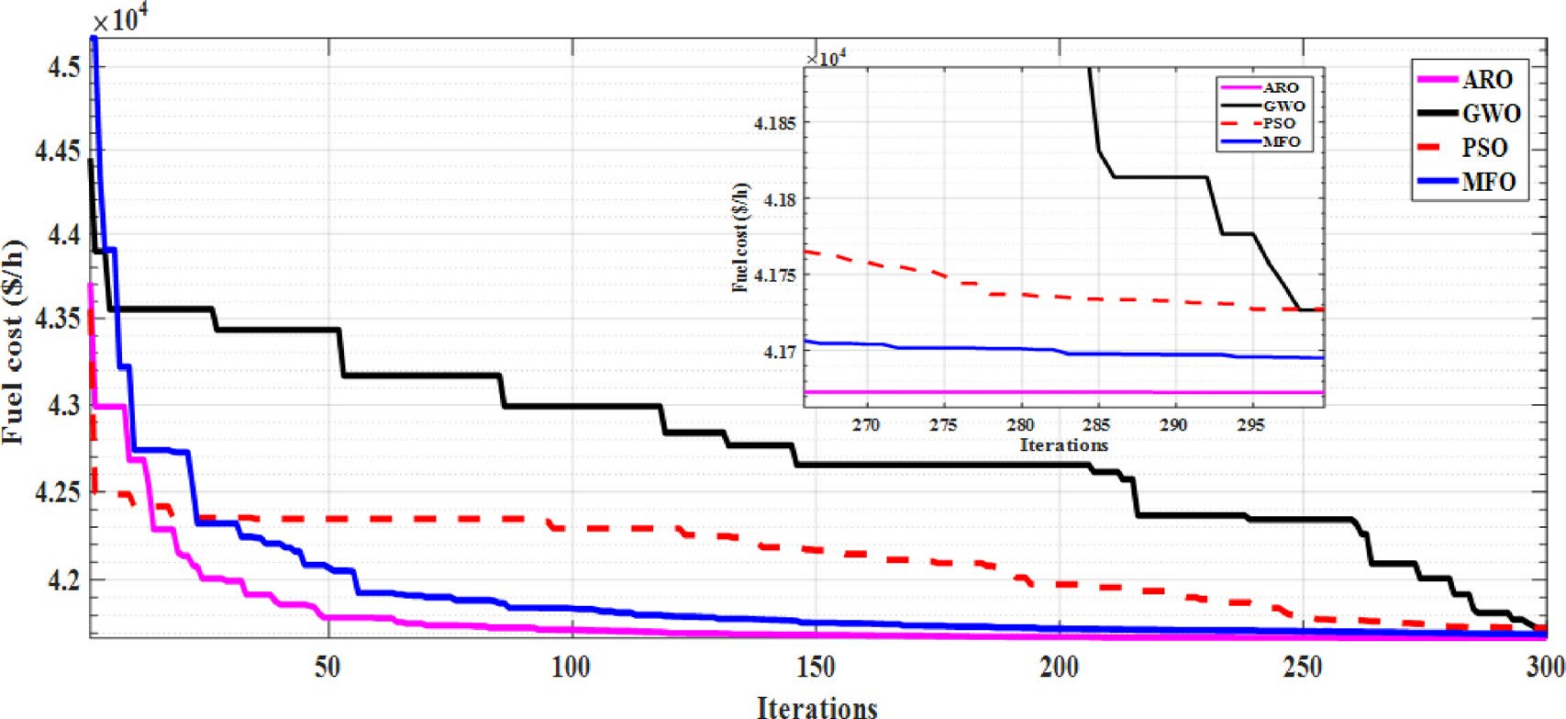
Rate of fuel cost convergence of Case 15 using ARO versus different methods.

Table 12 displays a comprehensive comparison between the proposed ARO algorithm and recent well-known algorithms in the literature. Additionally, Fig. 5 illustrates the convergence curves of ARO compared with three competitive methods in literature. Convergence curves clearly indicate that ARO exhibits a faster convergence rate than the others. Furthermore, Table 13 presents the statistical indices extracted from 50 runs of each algorithm specifically for Case 15. It is worth noting that ARO demonstrates the best fuel costs as well as lower STD, which serve as reliable indicators of the effectiveness of the proposed ARO algorithm.

**Table 12:** Simulation results of ARO and recent optimization methods for cases 15–18.

Case No.	OF	PSO	MFO	SFLA^58^	GSA^59^	ABC^44^	DA_PSO^49^	EMSA^54^	MSA^54^	ARO
15	FC	41,727.01	41,695.26	41,872.9	41,695	41,781	**41**,**674.6**	**41**,**666.2**	**41**,**673.59**	**41**,**672.88**
16	PL	**10.480**	11.295	-	-	12.626	**10.1212**	**-**	**-**	**9.076**
17	FC	**41**,**905.8**	41,792.5	-	-	-	**-**	**-**	**-**	41,689.29
	PL	**13.715**	13.914	-	-	-	**-**	**-**	**-**	**13.89**
18	FC	**42**,**031.22**	**42**,**010.03**	-	-	-	-	-	-	**41**,**703.31**
	PL	**16.949**	**15.713**	-	-	-	-	-	-	**14.356**
	VD	**1.579**	**1.392**	-	-	-	-	-	-	**0.967**

**Table 13:** Statistical indices of ARO in case 15 using different algorithms compared with ARO for 50 trials based on fuel costs minimization in $/h.

Algorithm	Max.	Min.	Average	Median	STD	Variance
GWO	42359.511	41726.484	41923.641	41,903.810	130.912	17,137.912
PSO	42396.201	41727.013	42014.195	42,018.992	179.887	32,359.361
MFO	42102.002	41695.265	41811.571	41,795.430	92.431	8543.453
ARO	41717.220	41672.890	41686.040	41,685.69	7.76	60.25

### Simulation results for large scale test systems

To validate the proposed ARO algorithm, the large-scale test systems are emulated in Cases 19–22 based on four systems with number of buses more than/equal 300 bus. These systems encompass power systems with varying numbers of buses. The primary data for the tested system was adapted from the MATPOWER 6.2 package^59^.

Table 13 presents the cost minimization of four cases, Cases 19–22, obtained by the ARO algorithm compared to those obtained by the MATPOWER 6.2 package for large scale power systems called IEEE 300-bus, the 1354pegase, 3012-bus, and 9241pegase test system. This comparison validates the scalability and efficiency of the ARO algorithm.

For the IEEE 300 bus, the fuel costs achieved using ARO amount to 508,324.16 $/h, whereas the MATPOWER package yields fuel costs of 719,692.27 $/h. The proposed ARO algorithm achieves a reduction of 29.34% in fuel costs compared to MATPOWER. In the case of the 1354pegase, the fuel costs OF using the proposed ARO equals 74,477.68 $/h $/h, while the MATPOWER 6.2 simulator yields fuel costs of 74,069.35 $/h. The proposed ARO algorithm reports a slight increase of 0.55% compared to MATPOWER.

For the third large-scale system, the ARO algorithm achieves fuel costs of 1,950,577 $/h, whereas the MATPOWER 6.2 simulator yields fuel costs of 2,591,706.57 $/h. The proposed ARO algorithm achieves a reduction of 24.73% in fuel costs compared to MATPOWER. Similarly, results are acquired for the 4th large-scale test system, the proposed ARO algorithm results in fuel costs of 166,233.12 $/h, while the MATPOWER 6.2 simulator reports fuel costs of 315,912.43 $/h with a reduction of 47.38%.

**Fig. 6:**
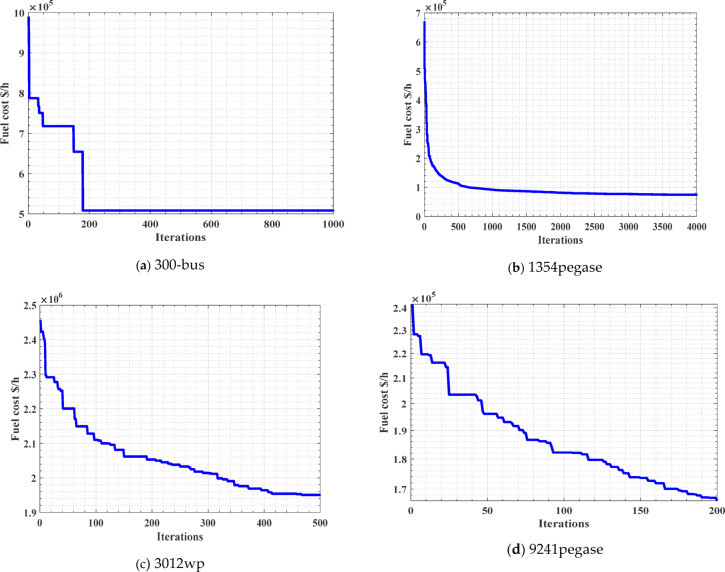
Convergence rates for large scale test systems.

Figures 6.a-6.d show the convergence curves for the four tested large-scale systems. Then, we can conclude that the proposed ARO has succeeded in achieving an economical solution, the economical reduction lies in the range of 24.73–47.38%, of the OFP even for most of the large-scale systems (Table 14).

**Table 14:** Costs reduction for large scale test grids by ARO and competitive methods cases 19–22.

Case No.	IEEE System Size	MATPOWER^59^	ARO	Reduction%
19	300-bus	719692.27 $/h	508324.16 $/h	29.34%
20	1354pegase	74069.35 $/h	74477.68 $/h	-0.55%
21	3012-bus	2591,706.57	1,950,577 $/h	24.73%
22	9241 pegase	315,912.430 $/h	166,233.12 $/h	47.38%

## Sensitivity analysis

**Fig. 7:**
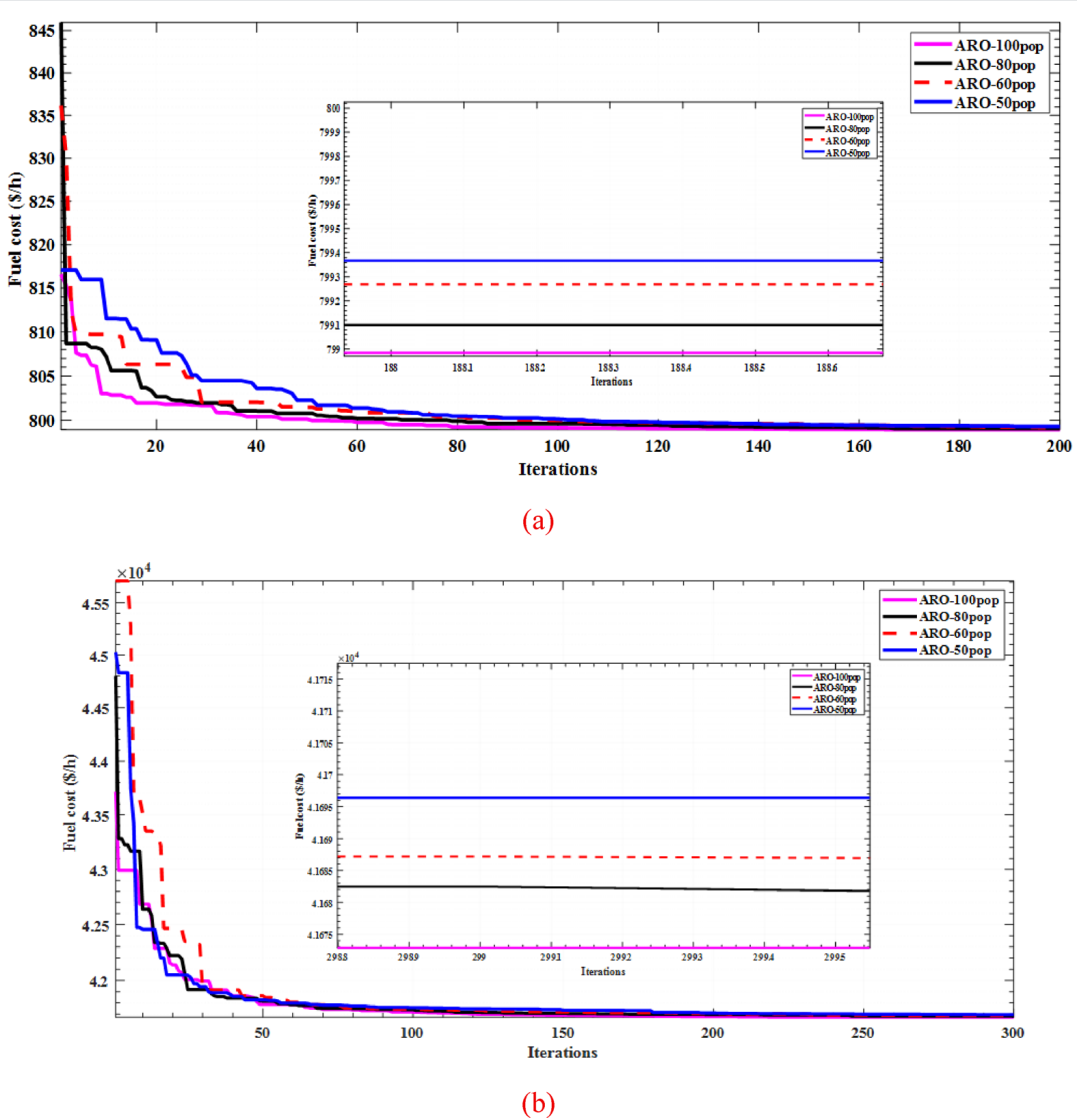
Convergence rates at different population sizes; (**a**) IEEE 30-bus system, (**b**) IEEE 57-bus system.

The sensitivity of the proposed ARO algorithm is carried out on the basic on the variation of population size and the maximum number of iterations. Two standard test systems, IEEE 30 bus and IEEE 57-bus, are selected as the most system used for solving OPF problem as benchmarking test systems. The developed simulation tested aim at minimization the fuel cost to explain the convergence characteristics with changing population size (P). Figure 7 (a) and (b) illustrates the sensitivity of convergence rate to population size. Additionally, statistics indices are reported with different number of iterations (Iter) by performing 50 independent runs with each variation. The sensitivity of ARO to maximum number of iterations is reported in Table 15 at the two test systems.

**Table 15:** Statistical indices of ARO with various numbers of iterations (50 times).

Metric	Min. of Fuel cost for IEEE 30 bus				Min. of Fuel cost for IEEE 57 bus			
Iteration	Iter = 50	Iter = 100	Iter = 150	Iter = 200	Iter = 100	Iter = 150	Iter = 200	Iter = 300
Best	800.25349	799.51297	799.51297	798.94372	41712.389	41692.749	41683.162	41672.886
Worst	802.98991	800.37915	800.37915	799.0327	41813.276	41773.893	41745.309	41717.223
Average	801.64471	799.90516	799.90516	798.98841	41754.495	41723.041	41708.025	41686.042
Variance	0.3435049	0.0555893	0.0555893	0.000377	664.4518	239.40763	175.82235	60.246767
Median	801.50106	799.92218	799.92218	798.98305	41752.955	41721.886	41705.937	41685.691
STD	0.5860929	0.2357738	0.2357738	0.0194162	25.776963	15.472803	13.259802	7.7618791

## Conclusions

This paper has successively solved the multi-dimensional OPF problem through finding the optimal operation settings associated with the power generators’ outputs. Single and multi-objectives frameworks have been considered. The objectives considered achieve the main power systems requirements involving technical and economic issues and respecting the limitation of environmental rules and bounds for emission-clean power grids. In this line, the Artificial rabbits’ optimization is devoted to finding the solution of the OPF problem. The effectiveness of the proposed algorithm is evaluated through a comprehensive comparison study with the existing works in literature. With six IEEE standard power systems, 22 different cases are implemented for testing the ARO performance in solving the OPF problem. Also, this paper extends its validation on different size large-scale test systems standard systems. It was proven that the routine of ARO has robust, and superior competitive performance compared with others at fine convergence rates. Significant technical and economic improvements are acquired as 24.73 -47.38% four large scale test systems. Numerical simulations have been implemented on small, medium, and large test systems and accentuate that ARO algorithm is superior in solving such complex OPF problems compared with other methods in the literature. Added to that, the ARO algorithm has a fast rate of convergence than other previous algorithms that make it an estimated algorithm in solving complex engineering problems. In future research, two aspects to be considered as dealing with various emergency events like N-1 contingency and preparing sufficient control actions to solve the impacts of these emergencies. In the second issue is to solve the OPF using upgraded optimization methods. Added to the previous issues, the future studies aim at incorporating uncertainties associated with renewable energy sources, load variations, and emerging new technologies such as electric vehicles and storage systems.

## Data Availability

All data generated or analyzed during this study are included in this published article.

## References

[1] Layth, A. B., Murtadha, A. K. & Jaleel, A. Solving optimal power flow problem using improved differential evolution algorithm. *Int. J. Electr. Electron. Eng. Telecommunications*. **11** (2), 146–155 (2022).

[2] Pan, X. et al. DeepOPF: A feasibility-optimized deep neural network approach for AC optimal power flow problems. *IEEE Syst. J.* **17** (1), 673–683 (2022).

[3] Elattar, E. E. & Salah, K. ElSayed. Modified JAYA algorithm for optimal power flow incorporating renewable energy sources considering the cost, emission, power loss and voltage profile improvement. *Energy* **178**, 598–609 (2019).

[4] Dokeroglu, T. et al. A survey on new generation metaheuristic algorithms. *Comput. Ind. Eng.* **137**, 106040 (2019).

[5] KS, G. D. Hybrid genetic algorithm and particle swarm optimization algorithm for optimal power flow in power system. *J. Comput. Mech. Power Syst. Control*. **2**, 31–37 (2019).

[6] Barakat, A. F., El-Sehiemy,Ragab, A. & Elsayd, Mohamed, I. Close accord on particle swarm optimization variants for solving Non-linear optimal reactive power dispatch problem. *Int. J. Eng. Res. Afr.* **46**, 88–105 (2020).

[7] Venkateswararao, B. & Balasubramanian, G. Whale optimization algorithm based optimal power flow: In view of power losses, voltage stability and emission. *In 2021 Innovations in Power and Advanced Computing Technologies (i-PACT)*, pp. 1–6. IEEE, (2021).

[8] Al-Bahran, L., Tawfeeq & Ali Qasim Abdulrasool. and. Multi objective functions of constraint optimal power flow based on modified ant colony system optimization technique. *IOP Conference Series: Materials Science and Engineering*. **1105** (1) IOP Publishing, (2021).

[9] Ettappan, M., Vimala, V., Ramesh, S. & Kesavan, V. T. Optimal reactive power dispatch for real power loss minimization and voltage stability enhancement using artificial bee colony algorithm. *Microprocess Microsyst.* **76**, 103085 (2020).

[10] Meng, A. et al. A high-performance crisscross search based on grey Wolf optimizer for solving optimal power flow problem. *Energy* **225**, 120211 (2021).

[11] Nellikkath, R. Physics-informed neural networks for optimal power flow. *Electr. Power Syst. Res.* **212**, 108412 (2022).

[12] Premkumar, M. et al. A reliable optimization framework using ensembled successive history adaptive differential evolutionary algorithm for optimal power flow problems. *IET Generation Transmission Distribution*. **17** (6), 1333–1357 (2023).

[13] Karboune, K. et al. Optimal power flow using firefly-algorithm. *Int. J. Dev.* **10** (1), 41–49 (2021).

[14] Alam, M. et al. The superiority of feasible solutions-moth flame optimizer using valve point loading. *Results Control Optim.* **17**, 100465 (2024).

[15] Sarda, J., Pandya, K., Kwang, Y. & Lee Hybrid cross entropy—cuckoo search algorithm for solving optimal power flow with renewable generators and controllable loads. *Optimal Control Appl. Methods*. **44** (2), 508–532 (2023).

[16] Shafik, M. B. et al. Optimal sizing and sitting of TCSC devices for multi-objective operation of power systems using adaptive seeker optimization algorithm. 2018 IEEE region ten symposium (tensymp). IEEE, (2018).

[17] Barakat, A. F. et al. Solving reactive power dispatch problem by using JAYA optimization algorithm. *Int. J. Eng. Res. Afr.* **36**, 12–24 (2018).

[18] Islam, M. et al. Marine predators algorithm for solving single-objective optimal power flow. *Plos One*. **16**, e0256050 (2021). 34383821 10.1371/journal.pone.0256050 PMC8360562

[19] Khan, A. et al. Solution of optimal power flow using non-dominated sorting multi objective based hybrid firefly and particle swarm optimization algorithm. *Energies* **13** (16), 4265. (2020). 10.1371/journal.pone.0235668 PMC7416925 32776932

[20] Abaza, A., Fawzy, A., El-Sehiemy, R. A., Alghamdi, A. S. & Kamel, S. Sensitive reactive power dispatch solution accomplished with renewable energy allocation using an enhanced Coyote optimization algorithm. *Ain Shams Eng. J.* **12**, 1723–1739 (2021).

[21] Abd, E. An improved version of salp swarm algorithm for solving optimal power flow problem. *Soft. Comput.* **25**, 4027–4052 (2021).

[22] Shaheen, Mohamed, A. M. et al. Solution of probabilistic optimal power flow incorporating renewable energy uncertainty using a novel circle search algorithm. *Energies* **15** (21), 8303. (2022).

[23] Chen, G. et al. Solving the multi-objective optimal power flow problem using the multi-objective firefly algorithm with a constraints-prior Pareto-domination approach. *Energies* **11** (12), 3438. (2018).

[24] Biswas, P. P. et al. Optimal power flow solutions using differential evolution algorithm integrated with effective constraint handling techniques. *Eng. Appl. Artif. Intell.* **68**, 81–100 (2018).

[25] Warid, W. Optimal power flow using the AMTPG-Jaya algorithm. *Appl. Soft Comput.* **91**, 106252 (2020).

[26] Khan, A. et al. Solution of optimal power flow using Non-Dominated sorting multi objective based hybrid firefly and particle swarm optimization algorithm. *Energies* **13** (16), 4265 (2020). 10.1371/journal.pone.0235668 PMC7416925 32776932

[27] Shaheen, A. M. et al. An enhanced optimizer of social network search for multi-dimension optimal power flow in electrical power grids. *Int. J. Electr. Power Energy Syst.* **155**, 109572 (2024).

[28] Han, Y., Qian, J. & Chen, G. Research of multi-objective modified Bat algorithm on optimal power flow problems. *Int. J. Syst. Control Inform. Process.* **3** (2), 150–171 (2020).

[29] Sehiemy, E. et al. A novel multi-objective hybrid particle swarm and salp optimization algorithm for technical-economical-environmental operation in power systems. *Energy* **193**, 116817 (2020).

[30] Nadimi-Shahraki, Mohammad, H. et al. Hybridizing of Whale and moth-flame optimization algorithms to solve diverse scales of optimal power flow problem. *Electronics* **11** (5), 831 (2022).

[31] Su, H. & Niu, Q. *Zhile Yang Optimal Power Flow. Using Improved Cross-Entropy Method Energies* **16**.14 : 5466. (2023).

[32] Naderi, E. et al. A novel hybrid self-adaptive heuristic algorithm to handle single-and multi-objective optimal power flow problems. *Int. J. Electr. Power Energy Syst.* **125**, 106492 (2021).

[33] Khunkitti, S., Siritaratiwat, A. & Premrudeepreechacharn, S. A Many-Objective marine predators algorithm for solving Many-Objective optimal power flow problem. *Appl. Sci.* **12** (22), 11829 (2022).

[34] Zellagui, M., Belbachir, N., Ragab, A. & El-Sehiemy Solving the optimal power flow problem in power systems using the mountain gazelle algorithm. *Engineering Proceedings* **56** (1), 176. (2023).

[35] Shaheen, A. M. et al. Multi-dimensional energy management based on an optimal power flow model using an improved quasi-reflection jellyfish optimization algorithm. *Eng. Optim.* **55** (6), 907–929 (2023).

[36] Sriram, K. et al. An extensive study using the beetle swarm method to optimize single and multiple objectives of various optimal power flow problems. *Int. Trans. Electr. Energy Syst.* **2023.1**, 5779700 (2023).

[37] Ahmadipour, M. et al. Optimal power flow using a hybridization algorithm of arithmetic optimization and aquila optimizer. *Expert Syst. Appl.* **235**, 121212 (2024).

[38] Jamal, R. et al. An improved pelican optimization algorithm for solving stochastic optimal power flow problem of power systems considering uncertainty of renewable energy resources. *Results Eng.* **26**, 104553 (2025).

[39] Akbari, E. et al. Optimal power flow via teaching-learning-studying-based optimization algorithm. *Electr. Power Compon. Syst.* **49**, 6–7 (2022).

[40] Pandya, S. & Hitesh, R. Jariwala. Single-and multiobjective optimal power flow with stochastic wind and solar power plants using moth flame optimization algorithm. *Smart Sci.* **10** (2), 77–117 (2022).

[41] Kahraman, H., Tolga, M., Akbel & Duman, S. Optimization of optimal power flow problem using multi-objective manta ray foraging optimizer. *Appl. Soft Comput.* **116**, 108334 (2022).

[42] Attia, A. F., El Sehiemy, R. A. & Hany, M. Hasanien. Optimal power flow solution in power systems using a novel Sine-Cosine algorithm. *Int. J. Electr. Power Energy Syst.* **99**, 331–343 (2018).

[43] Shaheen, Mohamed, A. M. et al. Probabilistic optimal power flow solution using a novel hybrid metaheuristic and machine learning algorithm. *Mathematics* **10**, 3036 (2022).

[44] Rezaei Adaryani, M. & Karami, A. Artificial bee colony algorithm for solving multi-objective optimal power flow problem. *Electr. Power Energy Syst.* **53**, 219–230 (2013).

[45] Shaheen, A. M., El-Sehiemy, R. A., Alharthi, M. M., Ghoneim, S. S. M. & Ginidi, A. R. Multi-objective jellyfish search optimizer for efficient power system operation based on multi-dimensional OPF framework. *Energy* **237**10.1016/j.energy.2021.121478 (2021).

[46] Babiker, A. et al. Optimal power flow: A review of State-of-the-Art techniques and future perspectives. In *IEEE Access*, vol. 13, pp. 60012–60039. 10.1109/ACCESS.2025.3556168 (2025).

[47] Wang, L. et al. Artificial rabbits optimization: A new bio-inspired meta-heuristic algorithm for solving engineering optimization problems. *Eng. Appl. Artif. Intell.* **114**, 105082 (2022).

[48] Khalil, A. et al. Enhancing the conventional controllers for load frequency control of isolated microgrids using proposed multi-objective formulation via artificial rabbits optimization algorithm. *IEEE Access.* **11**, 3472–3493 (2023).

[49] Shilaja, C. & Ravi, K. Optimal power flow using hybrid DA-APSO algorithm in renewable energy resources. *Energy Procedia*. **117**, 1085–1092 (2017).

[50] Bentouati, B., Chettih, S., Jangir, P. & Trivedi, I. N. A solution to the optimal power flow using multi-verse optimizer. *J. Electr. Syst.* **12**, 716–733 (2016).

[51] Bentouati, B., Chaib, L. & Chettih, S. A hybrid whale algorithm and pattern search technique for optimal power flow problem. In *Proceedings of the 8th International Conference on Modelling, Identification and Control* (ICMIC 2016), Algiers, Algeria, 15–17 November ; pp. 1048–1053. (2016).

[52] Ladumor, D. P., Trivedi, I. N., Bhesdadiya, R. H. & Jangir, P. Optimal power flow problems solution with STATCOM using meta-heuristic algorithm. In *Proceedings of the Third International Conference on Advances in Electrical, Electronics, Information, Communication and Bio-Informatics* (AEEICB 2017), Chennai, India, 27–28 February ; pp. 392–396. (2017).

[53] Ouafa, H., Linda, S. & Tarek, B. Multi-objective optimal power flow considering the fuel cost, emission, voltage deviation and power losses using Multi-Objective Dragonfly algorithm. In *Proceedings of the International Conference on Recent Advances in Electrical Systems*, Hammamet, Tunusia, 22–24 December (2017).

[54] Bentouati, B., Khelifi, A., Shaheen, A. M. & El-Sehiemy, R. A. An enhanced moth-swarm algorithm for efficient energy management based multi dimensions OPF problem. *J. Ambient Intell. Humaniz. Comput.* **12**, 9499–9519. 10.1007/s12652-020-02692-7 (2021).

[55] Ghasemi, M., Ghavidel, S., Ghanbarian, M. M., Gharibzadeh, M. & Azizi Vahed, A. Multi-objective optimal power flow considering the cost, emission, voltage deviation and power losses using multi-objective modified imperialist competitive algorithm. *Energy* **78**, 276–289. 10.1016/j.energy.2014.10.007 (2014).

[56] El-Sattar, S. A., Kamel, S., El Sehiemy, R. A., Jurado, F. & Yu, J. Single- and multi-objective optimal power flow frameworks using Jaya optimization technique. *Neural Comput. Appl.* **31**, 8787–8806 (2019).

[57] Ouafa, H., Linda, S. & Tarek, B. Multi-objective optimal power flow considering the cost, emission, voltage deviation and power losses using Multi-Objective Dragonfly algorithm. In *Proceedings of the International Conference on Recent Advances in Electrical Systems*, Hammamet, Tunusia, 22–24 December (2017).

[58] Khamees, A. K., El-Rafei, A., Badra, N. M. & Abdelaziz, A. Y. Solution of optimal power flow using evolutionary-based algorithms. *Int. J. Eng. Sci. Technol.* **9**, 55–68 (2017).

[59] Zimmerman, R. D. M.-S.C. Matpower [Software]. Available online: https://matpower.orgaccessed on jan (2023).

